# Diagnostic performance of FDG-PET/CT of post-transplant lymphoproliferative disorder and factors affecting diagnostic yield

**DOI:** 10.1007/s00259-019-04481-7

**Published:** 2019-08-24

**Authors:** F. M. Montes de Jesus, T. C. Kwee, X. U. Kahle, M. Nijland, T. van Meerten, G. Huls, R. A. J. O. Dierckx, S. Rosati, A. Diepstra, W. van der Bij, E. A. M. Verschuuren, A. W. J. M. Glaudemans, W. Noordzij

**Affiliations:** 1 Department of Nuclear Medicine and Molecular Imaging, University of Groningen, University Medical Center Groningen, Hanzeplein 1, Groningen, 9700 RB The Netherlands; 2 Department of Radiology, University of Groningen, University Medical Center Groningen, Groningen, The Netherlands; 3 Department of Hematology, University of Groningen, University Medical Center Groningen, Groningen, The Netherlands; 4 Department of Pathology and Medical Biology, University of Groningen, University Medical Center Groningen, Groningen, The Netherlands; 5 Department of Pulmonary Diseases and tuberculosis, University of Groningen, University Medical Center Groningen, Groningen, The Netherlands

**Keywords:** Post-transplant lymphoproliferative disorder, ^18^F-Fluoro-D-deoxyglucose positron emission tomography, FDG-PET/CT, Diagnosis

## Abstract

**Purpose:**

Post-transplant lymphoproliferative disorder (PTLD) is a serious complication after solid organ and hematopoietic stem cell transplantation, requiring a timely and accurate diagnosis. In this study, we evaluated the diagnostic performance of FDG-PET/CT in patients with suspected PTLD and examined if lactate dehydrogenase (LDH) levels, Epstein-Barr virus (EBV) load, or timing of FDG-PET/CT relate to detection performance of FDG-PET/CT.

**Methods:**

This retrospective study included 91 consecutive patients with clinical suspicion of PTLD and a total of 97 FDG-PET/CT scans within an 8-year period. Pathology reports and a 2-year follow-up were used as the reference standard. Diagnostic performance of FDG-PET/CT for detection of PTLD as well as logistic regression analysis for factors expected to affect diagnostic yield were assessed.

**Results:**

The diagnosis of PTLD was established in 34 patients (35%). Fifty-seven FDG-PET/CT scans (59%) were true negative, 29 (30%) were true positive, 6 (6%) false positive, and 5 (5%) false negative. Sensitivity of FDG-PET/CT for the detection of PTLD was 85%, specificity 90%, positive predictive value 83%, and negative predictive value 92%, with good inter-observer variability (*k* = 0.78). Of the parameters hypothesized to be associated with a true positive FDG-PET/CT result for the diagnosis of PTLD, only LDH was statistically significant (OR 1.03, *p* = 0.04).

**Conclusion:**

FDG-PET/CT has a good diagnostic performance in patients suspected of PTLD, with a good inter-observer agreement. Only LDH levels seemed to influence the detection performance of FDG-PET/CT. EBV-DNA load and timing of FDG-PET/CT after transplantation did not affect FDG-PET/CT diagnostic yield.

## Introduction

Post-transplant lymphoproliferative disorder (PTLD) is a serious complication after solid organ (SOT) and hematopoietic stem cell transplantation (HSCT). PTLD encompasses a heterogeneous morphologic spectrum, ranging from EBV driven polyclonal proliferations to aggressive monomorphic large B cell lymphomas. According to the World Health Organization, PTLD can be classified into 4 main groups: non-destructive lesions, polymorphic PTLD, monomorphic PTLD, and classical Hodgkin lymphoma PTLD [1].

PTLD has a variable clinical presentation that may include B-symptoms, lymphadenopathy, organ/allograft dysfunction, or a combination of non-specific symptoms. It is characterized by a bimodal presentation curve, with a peak incidence within 1 year after transplantation and a second peak after 4–5 years [2, 3]. However, the diagnosis of PTLD can be made at any time after transplantation and diagnostic work-up is usually initiated by the presence of B-symptoms and biochemical anomalies such as an increase in lactate dehydrogenase (LDH) and detectable Epstein-Barr virus (EBV) DNA. In PTLD, elevated LDH has been associated with a lack of response to initial therapy and worse prognosis [4–6]. EBV is recognized to play a crucial role in the immunopathogenesis of PTLD and EBV-DNA load monitoring is routinely performed for early detection of PTLD [7]. However, LDH and EBV-DNA load are non-specific for the detection of PTLD and particularly EBV-DNA load is not useful in EBV-negative PTLD which may compromise up to 50% of PTLD cases [8–10]. During diagnostic work-up, ^18^F-fluoro-2-deoxy-D-glucose (FDG) positron emission tomography (PET)/computed tomography (CT) may also be performed, allowing for whole-body visualization of metabolic active lesions and direct biopsy localization.

Although FDG-PET/CT is an established imaging modality in the detection of other FDG-avid lymphomas, with a reported median sensitivity of 90% and specificity of 91%, its diagnostic performance has not been extensively evaluated in PTLD [11, 12]. FDG-PET/CT may become an essential part of the diagnostic work-up in PTLD patients if proven to be of additional value for the detection of this disorder. However, biomarkers such as LDH, EBV-DNA load, and timing of FDG-PET/CT after transplantation may also influence its diagnostic yield. The purpose of this study was to evaluate the diagnostic performance of FDG-PET/CT in patients with suspected PTLD and to examine if LDH levels, EBV-DNA load, and timing after transplantation influence the detection performance of FDG-PET/CT.

## Materials and methods

### Study design and patients

This retrospective study included all consecutive patients between January 2010 and January 2019 for whom an FDG-PET/CT scan was requested. Indications for FDG-PET/CT requests are described in Table 1. The first FDG-PET/CT or in some patients the second scan after a 2-year negative follow-up period was included in the analysis (see “Reference standard”). Only patients 19 years and older were included. Patients with central nervous system involvement, complete tumor resection prior to FDG-PET/CT evaluation, and those without a biopsy or 2-year follow-up were excluded.

**Table 1:** Indications for FDG-PET/CT request*

	N (%)
Blood panel disturbances (e.g., complete blood count and biochemistry)	20 (20.6)
High EBV-DNA load	44 (45.3)
Physical symptoms (e.g., B-symptoms, enlarged lymph nodes, other non-specific symptoms)	37 (38.1)
Anomalies previous examination (e.g., colonoscopy, other non FDG-PET/CT imaging)	38 (39.2)

*Multiple indications possible for a single scan

### Patient record review

Relevant clinical and biochemical data were collected from the electronic patient files at the University Medical Center Groningen. These included age, gender, organ transplanted, time between transplantation and FDG-PET/CT, LDH levels, EBV-DNA load, and PTLD morphology and histology.

### FDG-PET/CT acquisition and interpretation

All FDG-PET/CT scans were performed on a Siemens Biograph 40 or 64 slice mCT (Siemens Healthineers, Erlangen, Germany) according to the European Association of Nuclear Medicine (EANM) procedure guidelines for tumor imaging [13]. Scans were performed after a minimum fasting time of 6 h. Images from the mid-thigh to skull base were acquired 60 min after intravenous administration of 3 MBq/kg FDG. Integrated FDG-PET/CT images were corrected for scatter and attenuation based on CT information. Scans were retrospectively reviewed by 3 readers (2 experienced nuclear medicine physicians (AG and WN) and 1 research fellow (FMJ) using syngo.via software (Siemens Healthineers, Erlangen, Germany). Readers reviewed the scans independently from each other and were blinded for other clinical/imaging findings and pathology results. Scans were considered positive for PTLD when FDG-avid lesions were present that could not be related to other pathology than PTLD. Scans were considered negative when no FDG-avid lesions suspicious for PTLD were found. Scans were considered equivocal when FDG-avid lesions were present, but this uptake could be due to either PTLD or due to other diseases/malignancy. In these cases, a differential diagnosis was noted. Discordant results between readers were re-evaluated in a consensus meeting and conclusively classified as positive or negative for PTLD.

### Reference standard

Pathology reports were used as a reference standard for PTLD diagnosis. Two experienced hematopathologists (SR, AD) were consulted for morphology clarification when necessary. In case of a PTLD-negative biopsy or lack of tissue for pathological examination, a 2-year follow-up period without pre-emptive PTLD therapy was accepted as the reference standard. Absence of lymphoma during this period has been shown to be an accurate marker for lack of disease in other lymphomas [14, 15]. True positive cases were defined as a PTLD-positive FDG-PET/CT and a PTLD-positive pathology result within 2 years after FDG-PET/CT. True negative cases were defined as a PTLD-negative FDG-PET/CT and no signs of PTLD within 2-year follow-up. False positive cases were defined as a PTLD-positive FDG-PET/CT and no signs of PTLD within a 2-year follow-up. False negative results were defined as a PTLD-negative FDG-PET/CT and pathology proven PTLD within a 2-year follow-up period.

### Statistical analysis

Baseline patient characteristics were summarized using medians with interquartile range (IQR) for non-normally distributed variables. Sensitivity, specificity, positive predictive value (PPV), and negative predictive value (NPV) of FDG-PET/CT for the detection of PTLD were calculated with a 95% confidence interval (CI). Logistic regression model analysis was carried out using mean serum LDH levels, mean EBV-DNA load (mean over a 31-day period before FDG-PET/CT), and time between transplantation and FDG-PET/CT with FDG-PET/CT result as a dependent variable. FDG-PET/CT results were dichotomized as true positive or not true positive (i.e., false positive, true negative, and false negative combined). The corresponding odds ratio (OR) and 95% CI were calculated. Statistical significance was set at *p* value ≤ 0.05. Inter-observer variability between the 3 observers was calculated using Fleiss kappa. The kappa value was interpreted according to the method of Landis and Koch: poor (0 to 0.20), fair (0.21 to 0.40), moderate (0.41 to 0.60), good (0.61 to 0.80), and perfect agreement (0.81 to 1) [16]. All statistical analyses were performed using SPSS, version 23.0 (IBM Corporation, Armonk, NY, USA).

## Results

### Patients

One-hundred-twelve potentially eligible patients were identified. Eleven patients were excluded due to central nervous system involvement, 6 due to complete tumor resection prior to FDG-PET/CT evaluation, and 4 due to lack of a reference standard. In total, 91 patients and 97 FDG-PET/CT scans were included in this study (Table 2). In 6 patients, 2 FDG-PET/CT scans were included because of PTLD suspicion on two different occasions with a time interval of more than 2 years. There were 50 males (55%) and 41 females (45%) with a median age of 54 years. The most frequently transplanted organ was the lung (*n* = 40, 44%) followed by the kidney (*n* = 31, 34.1%), liver (*n* = 11, 12.1%), HSCT (*n* = 4, 4.4%), multi-organ (*n* = 4, 4.4%), and the heart (*n* = 1, 1.1%). According to the reference standard, 34 patients (35%) were diagnosed with PTLD. There were 21 EBV-positive PTLDs (62%) and 13 EBV-negative PTLDs (38%). The median time between transplantation and FDG-PET/CT was 5 years (IQR; 9).

**Table 2:** Patient characteristics (*n* = 91)

Age at diagnosis (years)	
Median	54
Range	19–80
IQR	25
Gender	
Male	50 (55%)
Female	41(45%)
Transplanted organ	
Lung	40 (44.0%)
Kidney	31 (34.1%)
Liver	11(12.1%)
HSCT	4 (4.4%)
Multi-organ	4 (4.4%)
Heart	1 (1.1%)
Histology	
Non-destructive	2 (5.9%)
Polymorphic	6 (17.6%)
Monomorphic	24 (70.6%)
Classic Hodgkin type	1(2.9%)
Unclear	1(2.9%)
EBV status tumor	
Positive	21 (62%)
Negative	13 (38%)
Time between transplant and FDG-PET/CT (years)	
Median	5
Range	0–28
IQR	9

*IQR* interquartile range, *HSCT* hematopoietic stem cell transplantation

### Diagnostic performance of FDG-PET/CT

**Fig. 1:**
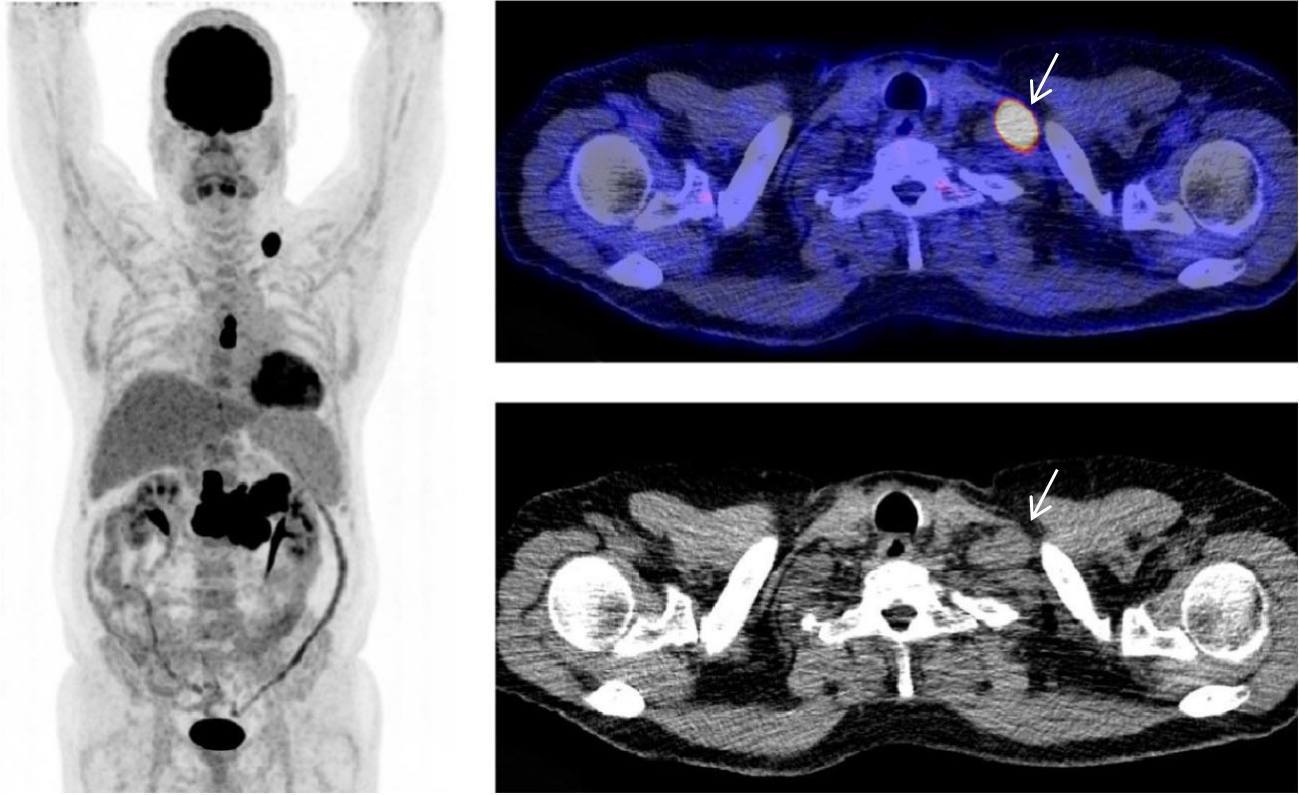
A 49-year-old male presented with low LDH levels (251 U/l) and low EBV DNA (1010 copies/ml) load, 6 years after lung transplantation. FDG-PET/CT was requested after palpable lymphadenopathy was clinically detected. Maximum intensity projection FDG-PET image shows metabolically active supraclavicular and mediastinal lymph nodes, and a large confluent abdominal lesion. Axial fused PET/CT (top right) and CT (bottom right) show the metabolically active supraclavicular lymph node, which proved to be monomorphic PTLD after biopsy

After a consensus meeting, the three readers assessed 35 scans to be positive for PTLD and 62 scans negative. Pathology confirmation was the reference standard in 64 scans (66%) and 2-year follow-up in 33 (34%). According to the reference standard, 57 scans (59%) were true negative, 29 (30%) were true positive, while 6 (6%) false positive, and 5 (5%) false negative results were observed (Table 3, Fig. 1). On a patient-based analysis, sensitivity of FDG-PET/CT for the detection of PTLD was 85%, specificity 90%, PPV 83%, and NPV 92% (Table 4).

**Table 3:** Classification of FDG-PET/CT scans (*n* = 97)

	PTLD present No. (%)	PTLD absent No. (%)
PET positive	29 (29.9)	6 (6.2)
PET negative	5 (5.1)	57 (58.8)

**Table 4:** Detection performance of FDG-PET/CT in PTLD

Analysis	Value %	95% CI
Sensitivity	85	68–94
Specificity	90	80–96
Positive predictive value	83	66–93
Negative predictive value	92	81–97
Accuracy	89	81–94

### False positive and false negative scans

**Fig. 2:**
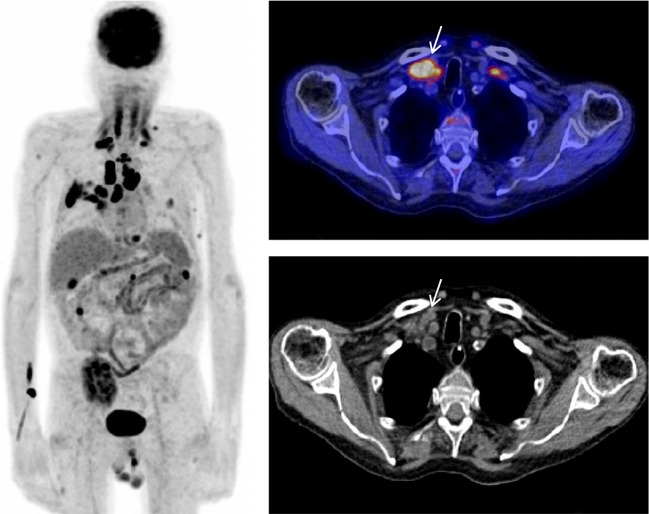
A 62-year-old male in which elevated LDH levels (347 U/l) and EBV-DNA load (1,032,500 copies/ml) were found after clinical monitoring within 1 year after kidney transplantation. FDG-PET/CT was subsequently requested. Maximum intensity projection FDG-PET shows disseminated metabolically active cervical, mediastinal, and lung parenchymal lesions with focal pararenal, native kidney, mesenteric, and liver lesions. Axial fused FDG-PET/CT (top right) and CT (bottom right) show a metabolically active supraclavicular lymph node that proved to be a granulomatous inflammation due to a mycobacterium after biopsy

In total, 6 scans were found to be false positive (Fig. 2). The final diagnoses of these false positive scans were as follows: 1 case of condyloma acuminata in the recto-uterine pouch, 1 case of an adenomatoid tumor in the round ligament, 1 case of small cell carcinoma in the transplanted lung, 1 case of systemic *Nocardia* infection, 1 case of *Aspergillus* infection in the lungs, and 1 case of spontaneous recovery of the suspected lesions without medical intervention, considered very unlikely to be PTLD. False positive results could be divided into two main categories: (1) other malignancies also showing high FDG uptake, making the differentiation between PTLD and other malignancy difficult and (2) infections, also taking up FDG and leading to a differential diagnosis of PTLD or infection. Five scans were concluded to be false negative: 2 cases with focal uptake in the tonsils/adenoid, interpreted as physiological uptake but confirmed to be non-destructive PTLD, 1 case with focal uptake in the rectum, interpreted as physiological uptake but confirmed to be polymorphic PTLD, 1 case with uptake in the lungs, interpreted as a primary lung tumor or infection but confirmed to be classic Hodgkin type PTLD, and 1 case considered to be a lung infiltrate without FDG-uptake but confirmed to be monomorphic PTLD (Table 5).

**Table 5:** Description false positive/negative cases

	Readers’ differential diagnosis*	Location FDG uptake	Final diagnosis/outcome
False positive (*n* = 6)	PTLD Pelvic malignancy	Recto-uterine pouch	Condyloma acuminata
	PTLD	Round ligament, intra-abdominal, retroperitoneal and inguinal lymph nodes	Adenomatoid tumor
	PTLD Liver abscess	Cervical, retroperitoneal lymph nodes, liver	Spontaneous recovery
	PTLD Disseminated infection	Supraclavicular, mediastinal, hilar and mediastinal lymph nodes, lung	Systemic (*Nocardia*) infection
	PTLD Lung malignancy	Lung	Small cell carcinoma
	PTLD	Mediastinal lymph nodes, lung	*Aspergillus* infection
False negative (*n* = 5)	Inflammation PTLD	Tonsils	Non-destructive PTLD tonsils
	Pneumonia Lung malignancy PTLD	Lung	Classic non-Hodgkin PTLD lung
	Physiologic uptake	Tonsils, adenoids	Non-destructive PTLD tonsils
	Physiologic uptake	Pelvis	Polymorphic PTLD rectum
	Unspecific lung infiltrate	No uptake	Monomorphic PTLD lung

*In order of most likely diagnosis

### Determinants of detection performance of FDG-PET/CT

According to univariate logistic regression, the only statistically significant parameter associated with a true positive FDG-PET/CT scan was serum LDH level with an OR of 1.03 (*p* = 0.04, 95% CI;1.001–1.06). Hence, for each 10 unit increase in LDH, the odds of having a true positive FDG-PET/CT scan were 3% higher. The remaining parameters, EBV-DNA load (OR; 1.0, *p* = 0.59, 95% CI;1.00–1.00), and time between transplant and FDG-PET/CT (OR; 1.05, *p* = 0.23, 95% CI;0.97–1.12) were not statistically significant in the univariate logistic regression analysis. For this reason, a multivariate logistic regression analysis was not performed (Table 6). Sub-analysis of only EBV-positive PTLD patients also revealed statistically insignificant results (OR; 1.0, *p* = 0.64, 95% CI;1.00–1.00).

**Table 6:** Association of parameters with a true positive FDG-PET/CT result for the diagnosis of PTLD

Parameter	Univariate OR	95% CI	*p*
Serum LDH levels	1.03	1.001–1.06	0.04
EBV-DNA load	1.00	1.00–1.00	0.59
EBV-DNA load (EBV-positive PTLD cases)	1.00	1.00–1.00	0.64
Time between transplant and FDG-PET/CT	1.05	0.97–1.12	0.23

### Inter-observer variability

From a total of 97 FDG-PET/CT scans evaluated prior to consensus, discordant results were reported in 14 scans. The majority of discordant results were due to FDG uptake in the lung parenchyma (*n* = 7). The differential diagnosis for these lesions included PTLD, primary lung tumor or infection. There were 5 cases of discordant results observed in the gastrointestinal tract and 1 discordant result for lesions in the pelvic area, in which observers reported difficulty in distinguishing between pathological and physiological uptake. Finally, 1 discordant finding, located in the adenoids of a young patient, was difficult to characterize as either pathological or physiological/infectious. From the 14 discordant FDG-PET/CT scans, 2 were false positive and 3 were false negative. The inter-observer variability was found to be good at *k* = 0.78 (95% CI; 0.68–0.88).

## Discussion

Although metabolic imaging with FDG-PET/CT has an established role in the diagnosis of non-Hodgkin and Hodgkin lymphoma, few studies have been carried out to assert its detection performance in PTLD [11, 17]. In our study population, compromising of 97 FDG-PET/CT scans in 91 patients with suspected PTLD, we found a sensitivity of 85%, specificity of 90%, PPV of 83%, and NPV of 92% with good inter-observer variability (*k* = 0.78). Of the determinants hypothesized to influence detection performance of FDG-PET/CT, only LDH levels were statistically significant with an OR of 1.03 (*p* = 0.04, 95% CI;1.001–1.06).

Current guidelines from the British Committee for Standards in Hematology, the British Transplantation Society, and National Comprehensive Cancer Network include no concrete recommendations on the use of FDG-PET/CT for diagnosing PTLD [18, 19]. The good diagnostic performance demonstrated in this study indicates that FDG-PET/CT is a valuable imaging modality for detecting PTLD. The good inter-observer variability also indicates that FDG-PET/CT interpretation was minimally reader dependent between two nuclear physicians and a junior reader. We identified common limitations associated with FDG-PET/CT false positive results such as inflammatory conditions and other malignancies in addition to false negative results in non-destructive and polymorphic PTLD, specific to this patient population. Because non-destructive lesions are commonly found in Waldeyer’s ring, such lesion location may be prone to be interpreted as physiological uptake [20, 21].

Although the majority of studies published on the diagnostic performance of FDG-PET/CT in PTLD are limited to case series, studies by Panagiotidis et al. and Dierickx et al. also reported good FDG-PET/CT diagnostic performance [22, 23]. Panagiotidis et al. included 40 patients with suspected PTLD and compared FDG-PET/CT diagnostic performance with CT. They concluded that FDG-PET/CT plays a significant role in the diagnosis of PTLD with high detection accuracy (sensitivity 88%, specificity 91%, PPV 88%, and NPV 91%) [22]. Some limitations of the study by Panagiotidis et al. included a smaller study population, half of that included in our study, and FDG-PET/CT scans were evaluated by a nuclear medicine physician and a radiologist without blinding for clinical data. Dierickx et al. investigated FDG-PET/CT diagnostic performance in 125 cases and reported high sensitivity of FDG-PET/CT in detecting PTLD (sensitivity 90%, specificity 89%, PPV 85%, and NPV 93%) [23]. However, their study was performed between 2003 and 2010 with 43% of the study population scanned with a stand-alone PET system and no information on the duration of follow-up in negative cases. A recent systematic review on the clinical performance of different imaging modalities in the diagnosis and treatment response evaluation of PTLD patients reported FDG-PET(/CT) to be the most frequently used imaging modality and a promising tool in this setting [24]. In the same review, false positive results were reported due to inflammatory conditions while false negative results occurred in areas of high physiological background activity and in non-destructive PTLD lesions. These results not only indicate the utility of FDG-PET/CT in PTLD patients but also concur with some of the findings in this study regarding potential causes for false positive and false negative results.

From the parameters hypothesized to be associated with a true positive FDG-PET/CT result for the diagnosis of PTLD, only LDH had a statistically significant odds increase. Uncontrolled proliferation of malignant cells with high cellular turnover is characterized by increased glycolysis and LDH release. High energy metabolism may translate into higher FDG uptake in tumor tissue and seemingly increase FDG-PET/CT detection performance [25]. Nevertheless, the influence of LDH levels on the detection performance of FDG-PET/CT should be confirmed in future research given the fact that the lower boundary of the 95% CI of the OR was close to 1 in the present study. The role of EBV in the pathogenesis of PTLD is well documented; however, EBV-DNA load was not associated with true positive FDG-PET/CT results in our study [26]. EBV monitoring is a common clinical practice for early PTLD detection and various studies have advocated its importance as a predictor of PTLD development [3, 27–31]. Nonetheless, EBV-DNA load does not seem to affect the detection performance of FDG-PET/CT. One potential explanation is the high percentage of EBV-negative PTLD in our study population, which may have decreased the clinical utility of the EBV-DNA load covariate. Yet, in a sub-analysis of only EBV-positive PTLD patients, we could not demonstrate a relationship between EBV DNA load and a true positive FDG-PET/CT either. We hypothesize that a single time cutoff value may not be indicative of imminent PTLD and consequently the need for FDG-PET/CT scanning. Instead, changes over time in EBV DNA load may yield more clinically relevant information as proposed by other studies [32, 33].

Due to the retrospective design of this study, information on immunosuppression adjustments was not available. As a consequence, it was not possible to evaluate EBV-DNA load changes over time as this is affected by immunosuppression intensity. PTLD patient population is inherently heterogeneous with regard to medical history, immunosuppression regimens, and treatment approaches. In our retrospective study, heterogeneity might have been introduced by the 8-year period inclusion time within various medical departments. Lack of standardization limits the analysis on how patient selection and timing of FDG-PET/CT affect its diagnostic performance, potentially inducing selection bias. Likewise, due to the retrospective nature of this study, we were not able to evaluate how different immunosuppressive regimens might have affected PTLD pathogenesis, which is of great clinical relevance and should be considered in future studies. Despite these limitations, the data presented reflects current clinical practice, as immunosuppression regimens are often department specific and there are currently no guidelines on the use FDG-PET/CT for the diagnosis of PTLD. Additional future studies may also focus on the role of interim and end-of-treatment FDG-PET/CT on survival, already explored by Keerberghen et al. and Zimmermann et al., and on the role of FDG-PET/CT in pediatric PTLD patients [34, 35]. Considering the marked differences in incidence, (imaging) presentation, and prognosis of the disease between children and adults, pediatric specific studies are warranted.

## Conclusion

FDG-PET/CT has a good diagnostic performance in patients with suspected PTLD. False positive results were due to other malignancies or infections while false negative results occurred in cases interpreted as physiological uptake (adenoids and rectum), other malignancy, and in 1 case of non-FDG-avid PTLD. Furthermore, a good inter-observer agreement was found, which further underlines the clinical utility of FDG-PET/CT. Only LDH levels seemed to influence the detection performance of FDG-PET/CT, while EBV-DNA load and time between transplant and FDG-PET/CT did not. The results of this study may help to implement FDG-PET/CT in future PTLD guidelines.

## Data Availability

The datasets used and/or analyzed during the current study are available from the corresponding author on reasonable request.

## Abbreviations

CI: confidence interval
CT: computed tomography
EANM: European Association of Nuclear Medicine
EBV: Epstein-Barr virus
FDG: ^18^F-fluoro-2-deoxy-D-glucose
HSCT: hematopoietic stem cell transplantation
IQR: interquartile range
LDH: lactate dehydrogenase
NPV: negative predictive value
OR: odds ratio
PET: positron emission tomography
PPV: positive predictive value
PTLD: post-transplant lymphoproliferative disorder
SOT: solid organ transplantation
